# Genome Sequence of the *Listeria monocytogenes* Food Isolate HPB913, Collected in Canada in 1993

**DOI:** 10.1128/genomeA.00911-16

**Published:** 2016-09-15

**Authors:** Arthur W. Pightling, Hugh Rand, Errol Strain, Franco Pagotto

**Affiliations:** a Listeriosis Reference Centre, Microbiology Research Division, Bureau of Microbial Hazards, Food Directorate, Health Canada, Ottawa, Ontario, Canada; b Center for Food Safety and Applied Nutrition, Biostatistics and Bioinformatics Branch, Office of Analytics and Outreach, U.S. Food and Drug Administration, College Park, Maryland, USA

## Abstract

*Listeria monocytogenes* is a pathogenic bacterium of importance to public health and food safety agencies. We present the genome sequence of the serotype 1/2a *L. monocytogenes* food isolate HPB913, which was collected in Canada in 1993 as part of an investigation into a sporadic case of foodborne illness.

## GENOME ANNOUNCEMENT

*Listeria monocytogenes* is a Gram-positive, pathogenic bacterium that is important to public health and food safety (1). *L. monocytogenes* can cause listeriosis when individuals with compromised immunities consume contaminated food (2, 3). Agencies tasked with securing the food supply are employing whole-genome sequencing for surveillance of *L. monocytogenes* in food and food processing facilities. Whole-genome sequence data have also proven useful for traceback investigations that seek to identify sources of sporadic and outbreak cases of listeriosis. In addition, researchers are utilizing comprehensive genome sequence analyses to investigate virulence factors that may influence the severity of listeriosis symptoms (4). Of particular interest are the serotype 1/2a strains of *L. monocytogenes*, which are commonly associated with listeriosis (5). We present the genome sequence of the serotype 1/2a *L. monocytogenes* food isolate HPB913, which was collected in Canada in 1993 as part of an investigation into a sporadic case of foodborne illness (National Center for Biotechnology Information [NCBI] BioSample no. SAMN03160826).

We generated Illumina sequence reads and submitted them to the NCBI Sequence Read Archive under accession number SRR1640166. We also assembled the reads with SPAdes v3.7.0 (6), using the BayesHammer error correction tool (7). This assembly yielded 82 contiguous sequences with 204.72-fold coverage. We removed one contiguous sequence from the final submission, as it is a run of adenine nucleotides. The submitted sequences have a combined length of 2,998,209 nucleotides and an *N*_50_ of 588,192 nucleotides. The largest sequence is 949,618 nucleotides in length. The GC content of all submitted sequences is 37.88 percent. The contiguous sequences were annotated with the NCBI Prokaryotic Genome Annotation Pipeline v3.3 (8). A total of 3,075 features were identified: 2,929 genes, 78 pseudogenes, one clustered regularly interspaced short palindromic repeat (CRISPR) array, seven rRNAs, 56 tRNAs, and four ncRNAs.

Pulsed-field gel electrophoresis indicates an AscI restriction digest pattern of LMACI.0001 and an ApaI pattern of LMAAI.0001. The ribotype pattern is 21-S-4 or DUP-1045. We also performed *in silico* multilocus sequence typing (9, 10), using the database provided by Institut Pasteur (http://bigsdb.pasteur.fr/listeria/listeria.html), and predicted the sequence type to be 120 (abcZ − *5, bglA − 6, cat − 2, dapE − 29, dat − 5, ldh − 3, lhkA − 1*) (11).

### Accession number(s).

This whole-genome shotgun project was deposited at DDBJ/EMGL/GenBank under the accession no. LZNK00000000. The version described in this paper is the first version, LZNK01000000.
